# Endoscopic full‐thickness resection of an esophageal leiomyoma located in close proximity to the azygos vein

**DOI:** 10.1002/deo2.30

**Published:** 2021-08-25

**Authors:** Hironari Shiwaku, Hiroki Okada, Akio Shiwaku, Keita Tanaka, Hideki Shimaoka, Kenji Maki, Fumihiro Yoshimura, Suguru Hasegawa

**Affiliations:** ^1^ Department of Gastroenterological Surgery Fukuoka University Faculty of Medicine Fukuoka Japan

**Keywords:** GIST, POEM, POET, SMT, third space endoscopy

## Abstract

Third‐space endoscopic techniques, such as peroral endoscopic tumor resection (POET) and submucosal tunneling endoscopic resection (STER), enable access to deep organs and tissues that have been previously inaccessible with an endoscope. We present a 29‐year‐old man with a submucosal tumor (40 × 25 mm) located at 5 o'clock in the upper thoracic esophagus. Histological diagnosis by endoscopic ultrasound–fine needle aspiration was leiomyoma. Computed tomography showed the azygos vein posterior to the tumor. However, because endoscopic ultrasound revealed space between them, POET was performed. Because the tumor originated from the deep layer of the muscularis propria, full‐thickness resection was performed to achieve R0 resection. The azygos vein arch was seen through the mediastinal space after tumor enucleation. The final histopathological diagnosis was leiomyoma. POET is a potentially revolutionary endoscopic technique that enables full‐thickness resection of nonepithelial lesions. Preoperative computed tomography or endoscopic ultrasound to determine peritumoral anatomy is important to ensure safety. During the procedure, it is important to operate under direct vision, accurately identify the tumor boundary, and dissect along the boundary to avoid damaging the tumor and surrounding structures.

## INTRODUCTION

Peroral endoscopic tumor resection (POET), or submucosal tunneling endoscopic resection (STER), is a new endoscopic technique that has the potential to revolutionize the treatment of submucosal gastrointestinal tract tumors as it enables full‐thickness resection of endoscopically accessible nonepithelial lesions irrespective of the site of origin.^1^, ^2^ We present a patient who underwent successful endoscopic full‐thickness resection of an esophageal leiomyoma located in close proximity to the azygos vein.

## CASE REPORT

**FIGURE 1:**
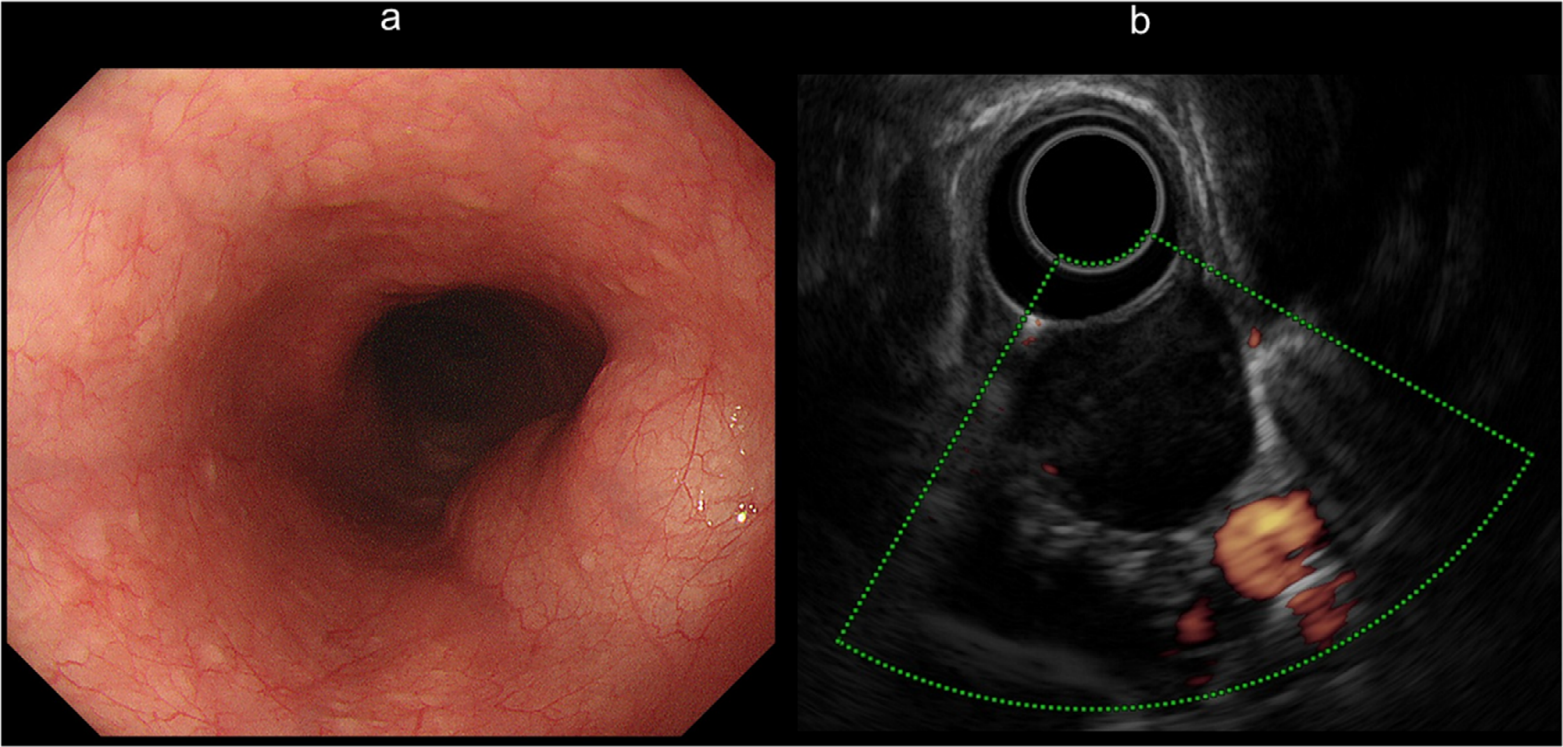
(a) Screening esophagogastroduodenoscopy showed a submucosal tumor located at 5 o'clock in upper thoracic esophagus. (b) Endoscopic ultrasound showed a uniform low‐echoic lesion with space between the tumor and azygos vein

**FIGURE 2:**
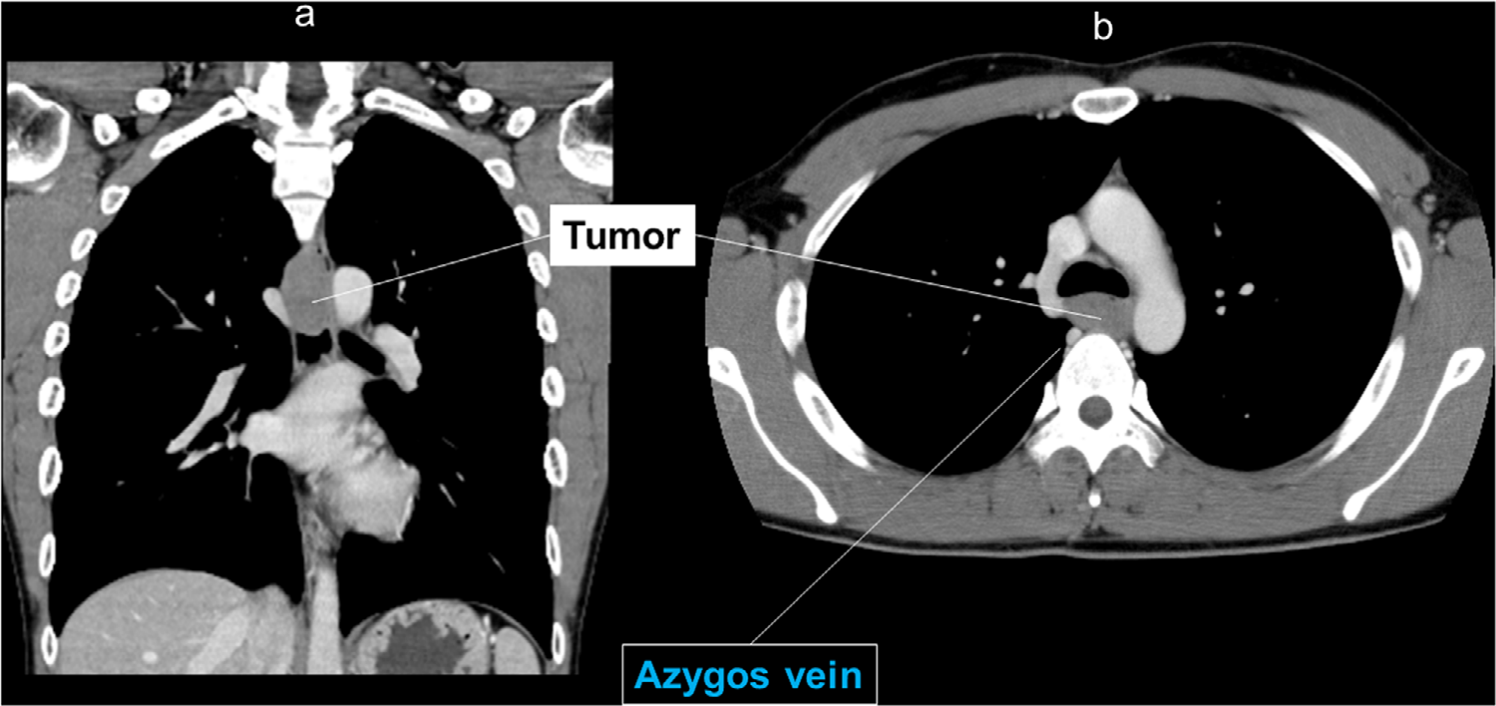
(a) Computed tomography showed a uniform low‐density mass in the mediastinum. Tumor size was 40 × 25 mm. (b) CT showed the azygos vein posterior to the tumor

**FIGURE 3:**
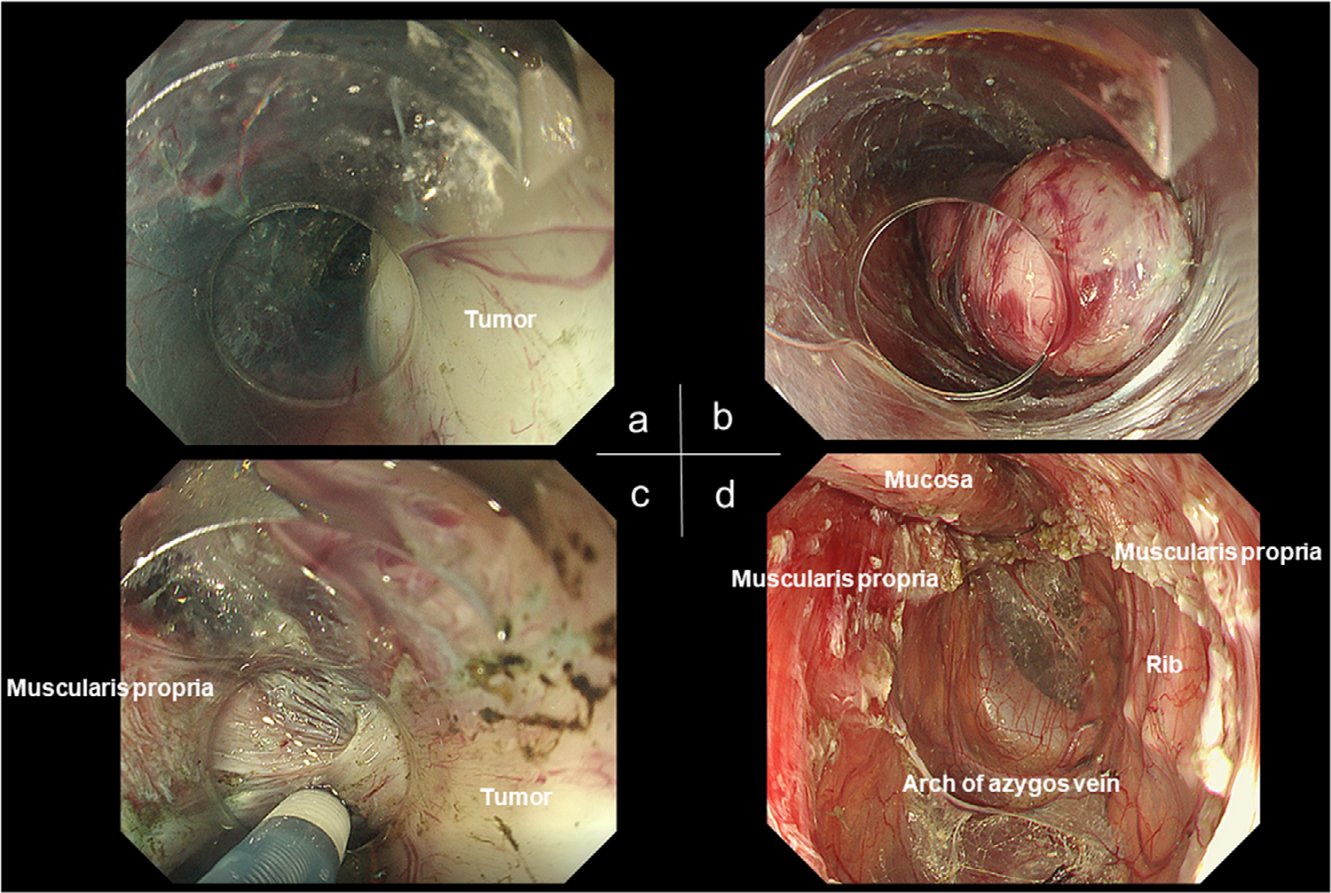
(a) During peroral endoscopic tumor resection, the tissue surrounding the tumor was dissected, except for the muscle layer side. (b) After dissection of tissue surrounding the tumor on the mucosal side. (c) As the tumor originated from the deep layer of the muscularis propria, full‐thickness resection was performed to achieve R0 resection. (d) The submucosal and mediastinal spaces after tumor enucleation. The azygos vein arch was seen through the mediastinal space

A 29‐year‐old man presented with an esophageal tumor detected by screening esophagogastroduodenoscopy. The tumor (40 × 25 mm) was submucosal and located at 5 o'clock in the upper thoracic esophagus (Figure 1a). Histological diagnosis by endoscopic ultrasound–fine needle aspiration (EUS–FNA) was leiomyoma. Computed tomography revealed that the azygos vein was posterior to the tumor (Figure 2). However, because there was space between them on EUS, we elected to perform POET (Figure 1b, Figure 3, and Supporting Information). POET was approved by the institutional review board of Fukuoka University Hospital (Approval number: T12‐11‐01) and written informed consent was obtained from the patient. The patient was treated in accordance with the principles of the Declaration of Helsinki.

POET was performed using an Olympus GIF‐H290T endoscope (Olympus Medical Systems, Tokyo, Japan). The Fujifilm DH‐33GR tapered distal attachment (Fujifilm Medical, Tokyo, Japan) was selected to allow deep insertion of the endoscope for direct vision without damaging the surrounding tissue. CO_2_ was used as the air supply from the endoscope. A Fujifilm FlushKnife BT (DK2620J) was used to perform electrocautery. The initial 2‐cm mucosal incision was made on the oral side of the tumor. The surrounding tissue was dissected from the tumor to create a submucosal pocket, leaving the muscle layer intact. Because the tumor originated in the deep layer of the muscularis propria, full‐thickness resection was performed to achieve R0 resection. Resection of the surrounding tissues was performed with the FlushKnife BT in cut mode to ensure that the tumor boundary was clearly visible. All manipulation was performed along the tumor to avoid injuring vital organs, especially the azygos vein. The tumor was resected safely en bloc and removed by a snare. The azygos vein arch was seen through the mediastinal space after tumor enucleation. The mucosal incision was closed tightly using the clip‐with‐line method to complete the procedure.^3^

**FIGURE 4:**
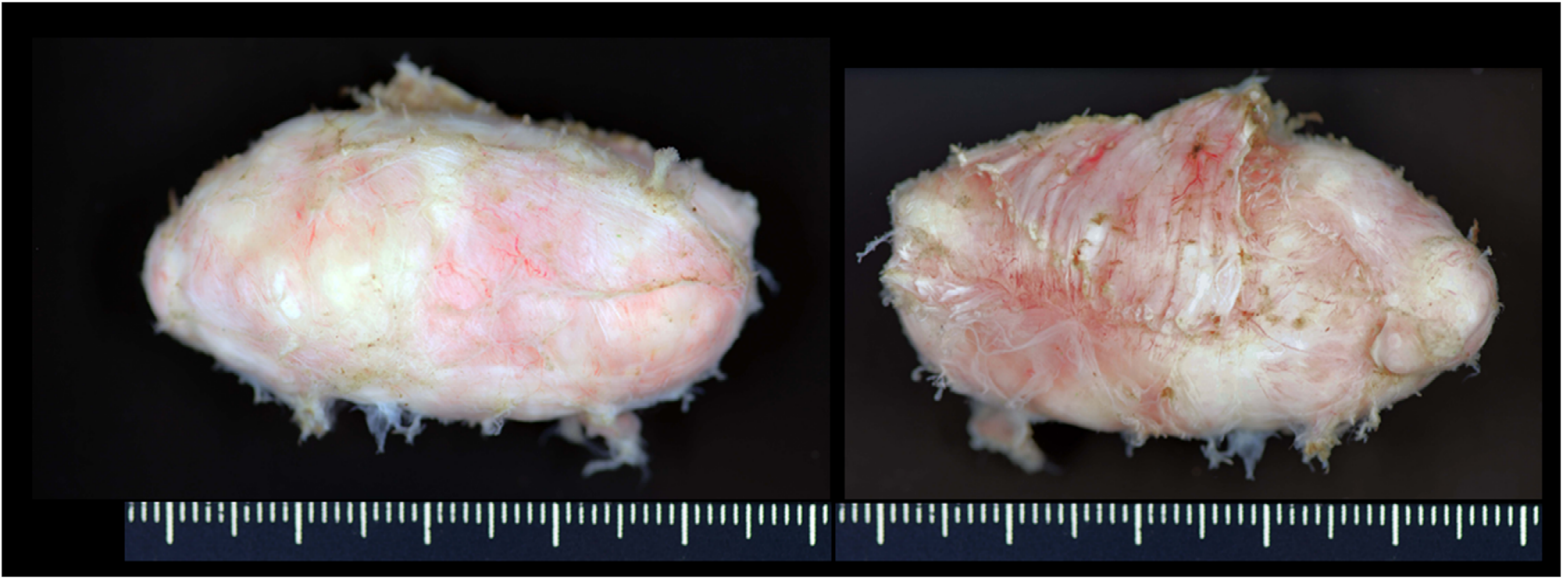
The back and front of the tumor. The muscle layer adheres to the back of the tumor. The histopathological diagnosis was leiomyoma

After the procedure, the patient experienced mild subcutaneous emphysema from the face to the inguinal region. He was discharged within a few days without major complications. The final histopathological diagnosis was leiomyoma (Figure 4).

## DISCUSSION

Before the introduction of peroral endoscopic myotomy (POEM), injury to the muscularis propria during endoscopic procedures was associated with serious adverse events, such as mediastinitis and peritonitis caused by perforation.^4^ Therefore, endoscopic resection was limited to lesions up to the submucosal layer; deeper tumors, including benign ones, were treated with surgical resection. However, based on the mucosal flap valve theory, POEM for esophageal achalasia showed that an endoscopic approach to deeper lesions is feasible provided that the mucosa is preserved.^5^ This knowledge broadened the potential applications of endoscopic treatment. The ability of POET to enucleate endoscopically accessible nonepithelial lesions irrespective of their site of origin has the potential to revolutionize treatment. POET is less invasive than surgery and scarless. Furthermore, tissues outside the gastrointestinal tract (such as the vagus nerve and lungs in this patient) are not touched, which preserves their function. However, during POET, it may become apparent that procedure completion is not possible (especially for large tumors); therefore, conversion to open surgery may be required. Clinicians should be prepared for surgical conversion and patients should be made aware of this possibility. Although there are no fixed indications for POET, Onimaru et al. reported that tumors with minor axis length >30 mm or tumor mass index (product of the major and minor axes) >1000 had a high probability of surgical conversion.^6^

A gastrointestinal stromal tumor (GIST) infrequently presents as a submucosal esophageal tumor (<5% of all GISTs).^7^, ^8^ Although surgical treatment is generally preferred for GISTs, enucleation of esophageal GISTs has been reported. Jiang et al. reported that complete enucleation for esophageal GISTs with an intact capsule and negative margins was feasible; however, tumors >5 cm in diameter and with mitotic count >5/50 high‐power fields should undergo esophagectomy because of their malignant behavior.^9^ Onimaru et al. reported that six patients with GIST pathologically classified as low risk underwent safe removal via POET and experienced favorable long‐term outcomes.^6^ Therefore, if en bloc resection can be performed in patients with low‐risk GISTs with an intact capsule, POET is safe and feasible. Histological diagnosis by preoperative EUS–FNA is mandatory to guide appropriate treatment.

Tumor damage during POET increases the risk of residual or recurrent tumor. Therefore, it is important to correctly identify the tumor boundary and dissect through the tissue outside the boundary. However, using coagulation mode during dissection frequently causes difficulty in identifying the boundary. Therefore, we use cut mode as much as possible. It is also important to operate under direct vision to avoid damaging surrounding structures. If POET is performed without inserting the endoscope deeply, there is a possibility that the operation will be partially blind. Deep insertion of the endoscope is sometimes difficult because the submucosal space is limited, especially in large tumors. Moreover, forcible deep insertion of the endoscope with a normal attachment can damage the mucosa or tumor capsule. Therefore, we selected a tapered distal attachment for deep insertion that allowed direct visualization without damaging the surrounding tissue. No blind manipulation was performed during the entire course of the procedure. Although the azygos vein was near, the tumor was safely resected without causing damage to the tumor or surrounding structures by manipulating along the tumor.

This case illustrates that third space endoscopy enables an approach to deep organs that have been previously inaccessible with an endoscope. With further device development, some treatments currently performed surgically may be performed less invasively with endoscopy in the future.

## CONCLUSION

POET is a potentially revolutionary endoscopic technique that enables full‐thickness resection of nonepithelial lesions. Preoperative computed tomography or EUS to determine peritumoral anatomy is important to ensure safety. During the procedure, it is important to operate under direct vision, accurately identify the tumor boundary, and dissect along the boundary to avoid damaging the tumor and surrounding structures.

## CONFLICT OF INTEREST

Hironari Shiwaku has received multicenter research grants from the Japanese Foundation for Research and Promotion of Endoscopy and a grant from the Japan Consortium for Advanced Surgical Endoscopy. Both grants are outside submitted work. The rest of the authors declare that they have no conflict of interest.

## FUNDING INFORMATION

None.

## Supporting information


**Video S1**. The procedure described in this report is summarized in the supporting video.

## References

[1] Inoue H , Ikeda H , Hosoya T , *et al*. Submucosal endoscopic tumor resection for subepithelial tumors in the esophagus and cardia. Endoscopy 2012; 44: 225–30. 22354822 10.1055/s-0031-1291659

[2] Xu MD , Cai MY , Zhou PH , *et al*. Submucosal tunneling endoscopic resection: A new technique for treating upper GI submucosal tumors originating from the muscularis propria layer (with videos). Gastrointest Endosc 2012; 75: 195–9. 22056087 10.1016/j.gie.2011.08.018

[3] Shiwaku H , Yamashita K , Inoue H , Hasegawa S . Closure of a mucosal entry using the clip‐with‐line method. Ann Gastroenterol 2018; 31: 252. 29507478 10.20524/aog.2018.0230 PMC5825961

[4] Inoue H , Minami H , Kobayashi Y , *et al*. Peroral endoscopic myotomy (POEM) for esophageal achalasia*. Endoscopy 2010; 42: 265–71. 20354937 10.1055/s-0029-1244080

[5] Sumiyama K , Gostout CJ , Rajan E , Bakken TA , Knipschield MA , Marler RJ . Submucosal endoscopy with mucosal flap safety valve. Gastrointest Endosc 2007; 65: 688–94. 17324411 10.1016/j.gie.2006.07.030

[6] Onimaru M , Inoue H , Bechara R , *et al*. Clinical outcomes of per‐oral endoscopic tumor resection for submucosal tumors in the esophagus and gastric cardia. Dig Endosc 2020; 32: 328–36. 31234231 10.1111/den.13471

[7] Rubin BP , Heinrich MC , Corless CL . Gastrointestinal stromal tumour. Lancet 2007; 369: 1731–41. 17512858 10.1016/S0140-6736(07)60780-6

[8] Hihara J , Mukaida H , Hirabayashi N . Gastrointestinal stromal tumor of the esophagus: Current issues of diagnosis, surgery and drug therapy. Transl Gastroenterol Hepatol 2018; 3: 6. 29441371 10.21037/tgh.2018.01.06 PMC5803007

[9] Jiang P , Jiao Z , Han B , *et al*. Clinical characteristics and surgical treatment of oesophageal gastrointestinal stromal tumours. Eur J Cardiothorac Surg 2010; 38: 223–7. 20206541 10.1016/j.ejcts.2010.01.040

